# *Panax Ginseng* Protects Against Doxorubicin-Induced Testicular Injury in Male Rats by Modulating NF-κB/COX-2 and AR Pathways

**DOI:** 10.1007/s12035-025-05116-9

**Published:** 2025-06-02

**Authors:** Yesim Yeni, Betul Cicek, Ahmet Hacimuftuoglu, Mustafa Ozkaraca, Behzad Mokhtare

**Affiliations:** 1 Faculty of Medicine, Department of Pharmacology, Malatya Turgut Ozal University, Battalgazi, 44210 Malatya, Turkey; 2 Faculty of Medicine, Department of Physiology, Erzincan Binali Yildirim University, Erzincan, Turkey; 3 Faculty of Medicine, Department of Medical Pharmacology, Ataturk University, Erzurum, Turkey; 4 Pathology Department, Faculty of Veterinary Medicine, Sivas Cumhuriyet University, Sivas, Turkey; 5 Pathology Department, Faculty of Veterinary Medicine, Dicle University, Diyarbakır, Turkey

**Keywords:** Doxorubicin, Male Fertility, Oxidative Stress, *Panax Ginseng*, Real-Time PCR

## Abstract

*Panax ginseng (PG)* is a medicinal plant used for many years to treat many diseases. The current study aimed to investigate the possible prophylactic and therapeutic effects of PG extract on doxorubicin (DOX)-induced testicular toxicity in rats. 32 adult male Sprague–Dawley rats (200–250 g) were used in the experiment. The experimental groups were designed as control (normal saline, intraperitoneal), DOX (18 mg/kg, intraperitoneal), PG (200 mg/kg, gavage), and PG + DOX (200 mg/kg, gavage). After treatment, serum levels of testosterone, interleukin-1β (IL-1β), glutathione (GSH), luteinizing hormone (LH), superoxide dismutase (SOD), lactate dehydrogenase (LDH), catalase (CAT), follicle stimulating hormone (FSH), tumor necrosis factor-α (TNF-α), and malondialdehyde (MDA) were measured. Then, gene expression, histopathological, and immunohistochemical analyses were performed on testicular tissues. Compared to DOX, treatment with PG + DOX showed a significant improvement in serum levels of FSH, testosterone, LH, TNF-α, IL-1β, MDA, SOD, LDH, GSH, and CAT. It was also observed that PG + DOX decreased nuclear factor-κB and cyclooxygenase-2 expression levels, increased androgen receptor expression, restored testicular histopathological structure, and significantly improved spermatogenesis. The results of the present study showed that PG may have an ameliorative effect against DOX-induced male reproductive toxicity, as DOX causes male reproductive toxicity. It can be concluded that PG is one of the effects that protect against DOX-induced testicular toxicity in rats by reducing lipid peroxidation and activating the antioxidant system. In light of this information, PG may be a useful agent to prevent the testicular toxicity observed in men receiving DOX treatment.

## Introduction

Testicular toxicity is a common consequence of chemotherapy agents used in the treatment of cancer and hematological malignancies [1]. Doxorubicin (DOX) is a potent anthracycline and anticancer agent used to treat hematological malignancies and solid tumors [2, 3]. Although successful in chemotherapy, treatment with DOX is limited due to its toxicity to various organs such as the kidney, brain, heart, and gonads [4, 5]. However, a major cause of DOX-induced toxicity is the increased production of reactive oxygen species (ROS) and oxidative stress [4, 6]. DOX cytotoxicity causes deoxyribonucleic acid degradation, lipid peroxidation, and ultimately apoptosis and necrosis in spermatocytes, an increase in abnormal sperm morphology, and a decrease in epididymal sperm count [7–9]. Therefore, searching for synthetic or natural antioxidant supplements to mitigate or prevent DOX-induced oxidative damage is important to ameliorate this drug-induced tissue toxicity [10, 11].

In recent years, numerous papers have shown that DOX-induced oxidative stress induces the production of proinflammatory cytokines such as nuclear factor kappa-B (NF-κB) [12, 13]. Evidence also suggests that the inflammatory response induced by DOX-induced NF-κB activation triggers the mitochondrial apoptosis pathway in cells [14]. In addition, DOX is known to induce one of the inflammatory enzymes, cyclooxygenase (COX) [15]. As it causes toxicity in the testicular tissue, this may lead to impaired spermatogenesis. The male sex hormones, androgens, exert their biological effects in a variety of target organs, and most of the biological effects of androgens are mediated by transcriptional regulation through activation of the nuclear androgen receptor (AR) [16]. ARs are transmembrane proteins of androgen hormones and are found in a variety of tissues. DOX is an antineoplastic agent with an affinity for ARs and can cause toxicity to testicular tissue. This toxicity can cause side effects such as decreased sexuality and infertility in men by acting on androgen receptors [17]. Therefore, testicular tissue and androgen receptors should be considered when using such drugs. In addition, available information shows that exposure to DOX leads to extreme production of lipid peroxidation. Furthermore, DOX-induced oxidative stress triggers apoptotic cascades via extrinsic and intrinsic signaling pathways and includes suppression of antioxidant enzyme activity [18, 19].

The primary active constituent of red ginseng responsible for the pharmacological effects of *Panax ginseng* (PG) is ginsenosides. Recently, evidence from in vitro and in vivo work has shown that ginsenosides improve endothelial dysfunction by normalizing myocardial oxidative stress [20]. In addition, results from initial clinical work in healthy subjects demonstrated the beneficial effects of ginseng supplementation in reducing oxidative stress by upregulating the activity of antioxidant enzymes [21]. Oxidative stress is a condition that occurs due to the action of free radicals produced in the body, and this is often seen in testicular tissue. In particular, Leydig cells, which are involved in synthesizing the hormone testosterone, can be damaged under oxidative stress [22]. Therefore, anti-inflammatory and antioxidant properties of PG may exert an oxidative stress-reducing effect on the testicular tissue, which may prevent damage to Leydig cells. However, these properties of PG have not been adequately investigated, and further research is needed. Here, authors aimed to examine the role of PG in DOX-induced testicular toxicity at the histological, biochemical, and molecular levels. To this end, immunohistochemical and real-time PCR analyses were performed to evaluate the effects of PG on the gene expressions of NF-κB, AR, and COX-2. Additionally, histopathological and biochemical analyses were performed to investigate the pathological changes in testicular tissue and their effects on oxidative stress markers and sex hormones in serum.

## Materials and Methods

### Chemicals

DOX (10 mg/ml Doxorubicin HCI®) was purchased from Koçak Farma (Turkey). PG (100 mg softgel capsules, 10% Ginsenosides) was purchased from Herb Pharm (https://tr.iherb.com/pr/natural-factors-herbalfactors-panax-ginseng-100-mg-60softgels/2631). The PG softgel capsule was cut using scissors to remove the contents. Distilled water was added, and the solution was warmed for 10 min in a 45 °C water bath. It was then subjected to ultrasonication to prepare a homogenized solution.

### Animals

The current study was approved by the Atatürk University Animal Experiments Local Ethics Committee (No: E-42190979–000–2200417010), and all stages of the study comply with ethical rules. 32 adult male Sprague–Dawley rats (200–250 g; 6–8 weeks old) were obtained from Atatürk University Medical Experimental Research and Application Centre. The rats were housed in pathogen-free cages (23 ± 1 °C and 12/12 h light/dark cycle) with free access to rodent chow pellets and water. Rats were acclimatized for 7 days before the experiments began.

### Experimental Design

**Fig. 1:**
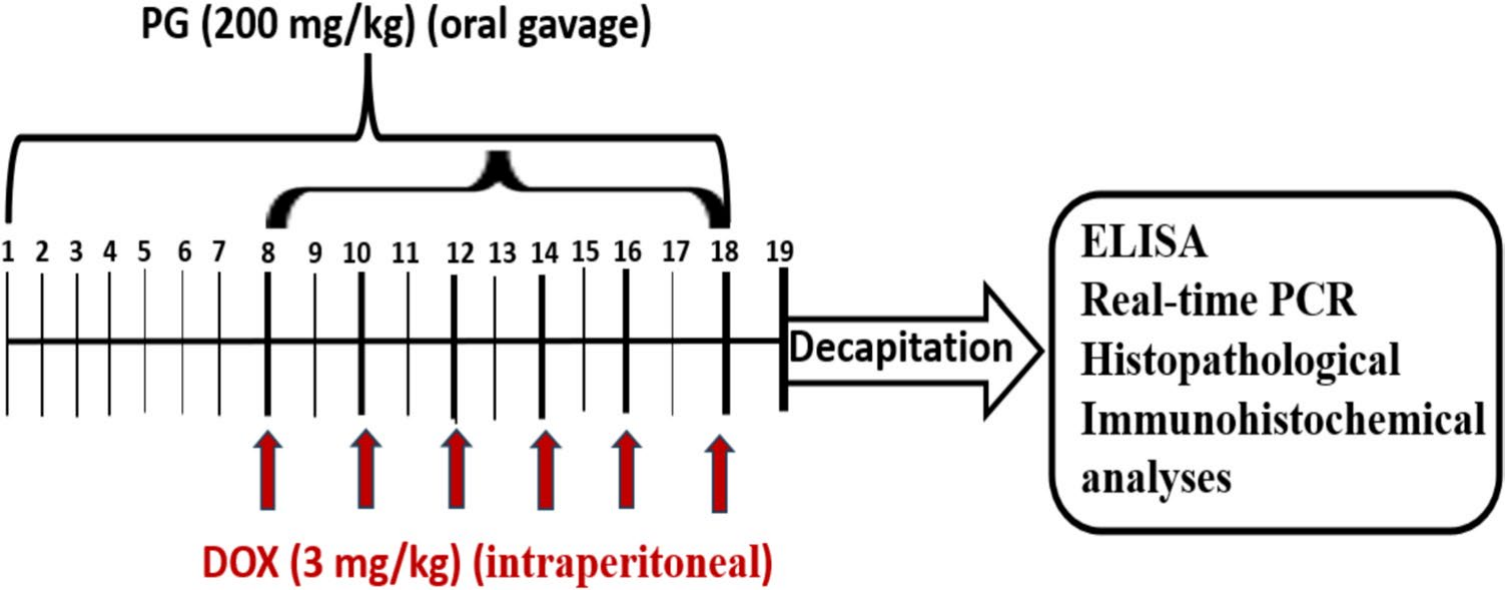
Schedule of the study's experimental procedures

After a 7-day adaptation period, the rats were randomly divided into four groups (eight rats each). Group 1 (control); rats received intraperitoneal normal saline on days 8, 10, 12, 14, 16 and 18; Group 2 (DOX): A total cumulative dose of DOX (18 mg/kg) was administered by intraperitoneal injection in six separate doses of 3 mg/kg on days 8, 10, 12, 14, 16 and 18 [23]; Group 3 (PG): PG 200 mg/kg/day was administered by oral gavage for 18 days; Group 4 (PG + DOX): Before starting DOX injection, PG was administered at a dose of 200 mg/kg/day by oral gavage for a week and continued to be administered simultaneously with DOX (on days 8, 10, 12, 14, 16, and 18) for 11 days [24]. The experiment was terminated on day 19. In Fig. 1, the timeline illustrates animal treatments, decapitation planning, biochemical, real-time PCR, histopathological, and immunohistochemical analyses.

5 ml blood samples were collected by cardiac puncture from rats anesthetized with an intraperitoneal injection of thiopental sodium (50 mg/kg) (PentalⓇ IE Ulagay, Turkey). After blood collection, the testicular tissues of the rats were quickly dissected, and the rats were euthanized by cervical dislocation. Blood samples were centrifuged at 4000 rpm for 10 min at 4 °C and stored at −80 °C to obtain serum. Dissected testicular tissue was stored in an RNA stabilization reagent for molecular analysis. In addition, testicular tissues were fixed in 10% neutral buffered formalin for immunohistochemical and histopathological studies. The right testis was used for molecular analyses, while the left testis was used for pathological examinations.

### Biochemical Analysis

The authors used the ELISA method to detect oxidant/antioxidant and inflammatory markers in the serum obtained. In this respect, interleukin-1β (IL-1β), glutathione (GSH), testosterone, superoxide dismutase (SOD), lactate dehydrogenase (LDH), luteinizing hormone (LH), tumor necrosis factor-α (TNF-α), Follicle stimulating hormone (FSH), catalase (CAT) and malondialdehyde (MDA) (Elabscience, USA) activities were prepared according to the instructions of each commercially available kit. Optical density was measured at 450 nm using an ELISA plate reader (Bio-Tek Instruments, USA).

### Testis Tissue RNA Isolation and cDNA Synthesis

Samples were stored at −80 °C until RNA isolation. A high-purity RNA isolation kit (Roche, Basel, Switzerland) was used for RNA isolation using the Rotor-Gene Q instrument (QIAGEN). The RNA samples obtained were then converted into cDNA using the first strand of the Transcriptor cDNA Synthesis Kit (Roche, Basel, Switzerland). The cDNA samples were then measured by nanodrop spectrophotometry, and the resulting cDNA was stored at −20 °C.

### Real-Time PCR Analysis

The relative expression levels of NF-κB, AR, and COX-2 in right testicular tissue were determined using Rotor-Gene Q. The expressions of NF-κB, AR, COX-2, and β-actin were examined by real-time PCR using the following primers: NF-κB: F, 5'-TGAACCGAAACTCTGGCAGCTG-3'NM_021975 R, 5'-CACAGCGACCATCAATAGCA-3'; AR: F, 5'-ATGGTGAGCAGAGTGCCCTATC-3'NM_000044 R, 5'-ATGGTCCCTGGCAGTCTCCAAA-3'; COX-2: F, 5'-GCGACATACTCAAGCAGGAGCA-3'NM_011198 R, 5'-AGTGGTAACCGCTCAGGTGTTG-3'; β-actin: F, 5'-CACCATTGGCAATGAGCGGTTC-3'NM_001101 R, 5'-AGGTCTTTGCGGATGTCCACGT-3'. The data obtained were analyzed as fold changes using the 2^−ΔΔCt^ method [25].

### Histopathological Analysis

Left testes were immediately removed and fixed in 10% neutral buffered formalin for 24–48 h. Fixed testicular tissues were routinely handled, and tissues were embedded in paraffin and serially sectioned at 5 μm thickness. All tissue sections were stained with hematoxylin and eosin for histopathological evaluation before being examined under a light microscope [26]. Subsequently, 10 randomly selected tubules were examined at 40 × magnification (Leica, DM2500). Scoring was performed using Johnsen's mean testis biopsy score criteria to assess (Table 1) [27].

**Table 1:** Mean testis biopsy scores (MTBS)

Score	Description
1	No cells
2	Sertoli cells without germ cells
3	Only spermatogonia
4	Only a few spermatocytes
5	Many spermatocytes
6	Only a few early spermatids
7	Many early spermatids without differentiation
8	Few late spermatids
9	Many late spermatids
10	Full spermatogenesis

### Immunohistochemical Studies

Testicular tissues fixed in 10% neutral buffered formalin (control and treatment groups) were embedded in paraffin blocks after routine alcohol-xylol processing. 5 µm sections taken on polylysine slides were passed through the xylol-alcohol series, washed with phosphate-buffered saline, and incubated in 3% H_2_O_2_ for 10 min. The tissues were treated with antigen retrieval solution (Citrate Concentrated Solution, Santa Cruz, Cat No: sc-294091) at 500 watts for 2 × 5 min to release the antigen. The primary antibodies used were selected to be compatible with rat tissue. The tissues were then washed with phosphate-buffered saline and incubated with Androgen receptor Polyclonal Antibody (Bioss, Cat No:bs-0118R), Rabbit polyclonal to COX2/Cyclooxygenase 2 (Abcam, Cat No: ab 15191) and Rabbit monoclonal [E381] to NF-κB (Abcam, Cat No: ab7971) primary antibodies at a dilution ratio of 1/150 at + 4ºC overnight. For the negative control, the primary antibody was omitted during immunohistochemical staining. Secondary large volume detection system: anti-polyvalent, HRP (Thermo Fischer, Cat No: TP-125-HL) was used as suggested by the manufacturer. The chromogen used was 3,3′-diaminobenzidine. After counterstaining with Mayer's hematoxylin, sections were coverslipped with entellan and examined by light microscopy (Leica, DM2500). Immunopositivity was graded as none (-), mild (+), moderate (+ +), severe (+ + +), and very severe (+ +  + +).

### Statistical Analysis

Quantitative data were expressed as mean ± standard deviation. Tukey's LSD test was used after one-way ANOVA to evaluate the results obtained in the biochemical and molecular data. The Kruskal–Wallis method was used to assess the intensity of immunoreactivity between non-parametric groups. Analysis was performed using SPSS 22.0 (IBM, USA). Results less than or equal to *p* < *0.05* were considered statistically significant.

## Results

### Effect of PG Treatment on DOX-Induced Oxidative Stress

**Fig. 2:**
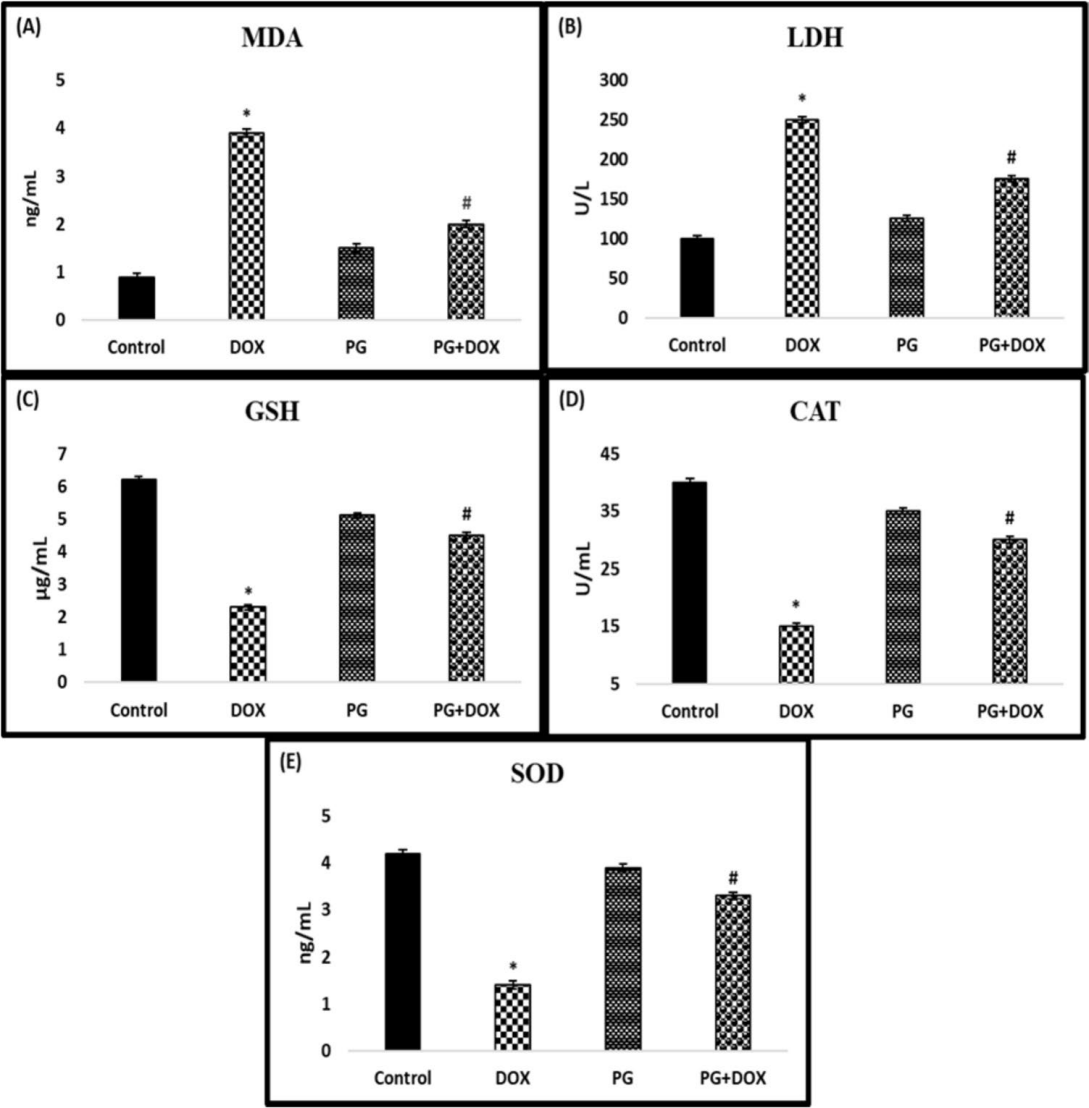
Effects of PG on antioxidant enzymes and lipid peroxidation. Detecting the level of (A) MDA, (B) LDH, (C) GSH, (D) CAT, and (E) SOD. *p < *0.05* versus. Control group, #*p* < *0.05* versus. DOX group (*n* = 8). Statistical analysis was done by one-way ANOVA followed by Tukey's LSD test. DOX: Doxorubicin, PG: 200 mg/*kg Panax ginseng*, DOX + PG: Doxorubicin + 200 mg/kg *Panax ginseng*

To determine whether PG alleviates DOX-induced oxidative damage in rats, LDH, SOD, CAT, and GSH activity and MDA content in rat serum were evaluated. In rats injected with DOX as shown in Fig. 2, serum CAT, SOD, and GSH activities were significantly reduced, while MDA and LDH activities were increased compared to the control group (*p* < *0.05*). SOD, CAT, and GSH activities were increased whereas LDH and MDA activities were significantly decreased in the PG + DOX group compared to the DOX group (*p* < *0.05*).

### Effect of PG Treatment on DOX-Induced Proinflammatory Cytokines

**Fig. 3:**
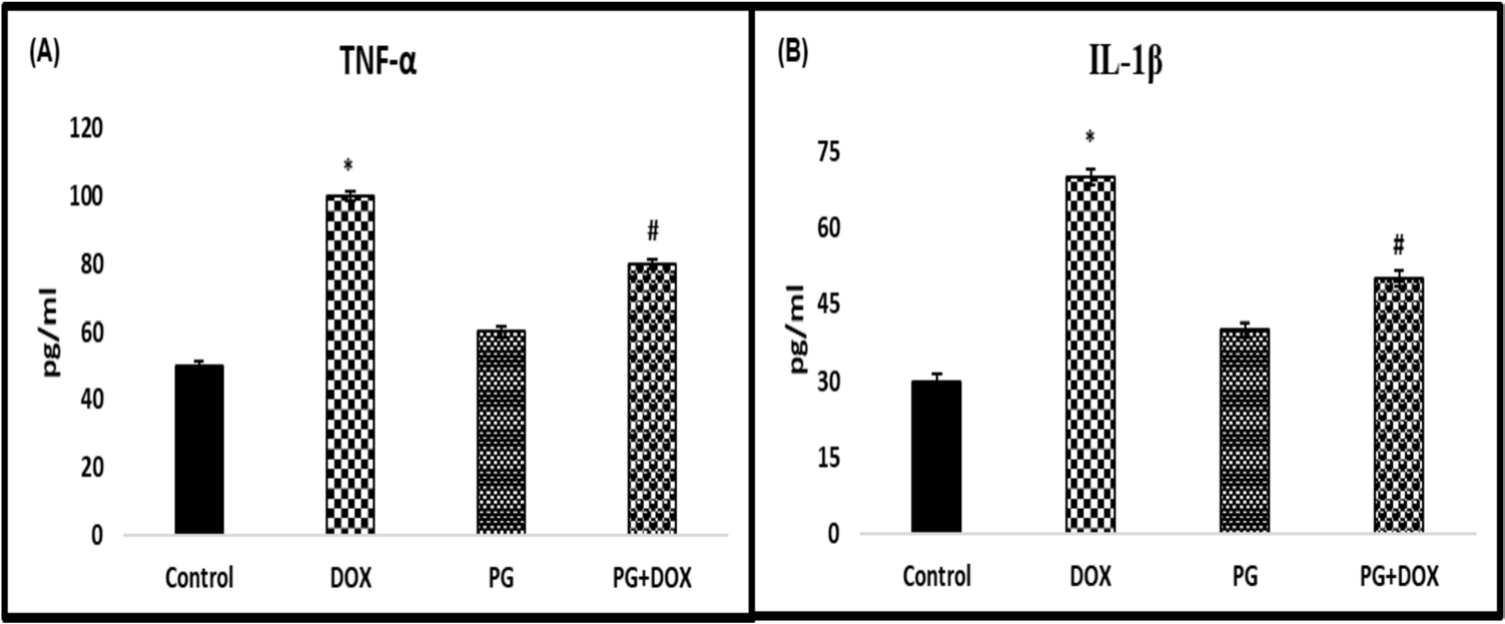
Effects of PG on proinflammatory cytokines. Detecting the level of (A) TNF-α and (B) IL-1β. *p < *0.05* versus. Control group, #*p* < *0.05* versus. DOX group (*n* = 8). Statistical analysis was done by one-way ANOVA followed by Tukey's LSD test. DOX: Doxorubicin, PG: 200 mg/kg *Panax ginseng*, DOX + PG: Doxorubicin + 200 mg/kg *Panax ginseng*

The potential anti-inflammatory effect of PG against DOX-induced proinflammatory response was investigated by evaluating TNF-α and IL-1β biomarkers in rat serum. A significant increase in TNF α and IL-1 β levels was observed in DOX-injected rats compared to control rats (*p* < *0.05*). Treatment with PG + DOX significantly inhibited the DOX-induced increase in serum pro-inflammatory cytokine levels compared to DOX treatment alone (*p* < *0.05*) (Fig. 3).

### PG Increases Levels of Sex Hormone in DOX-Induced Testis Damage in Rats

**Fig. 4:**
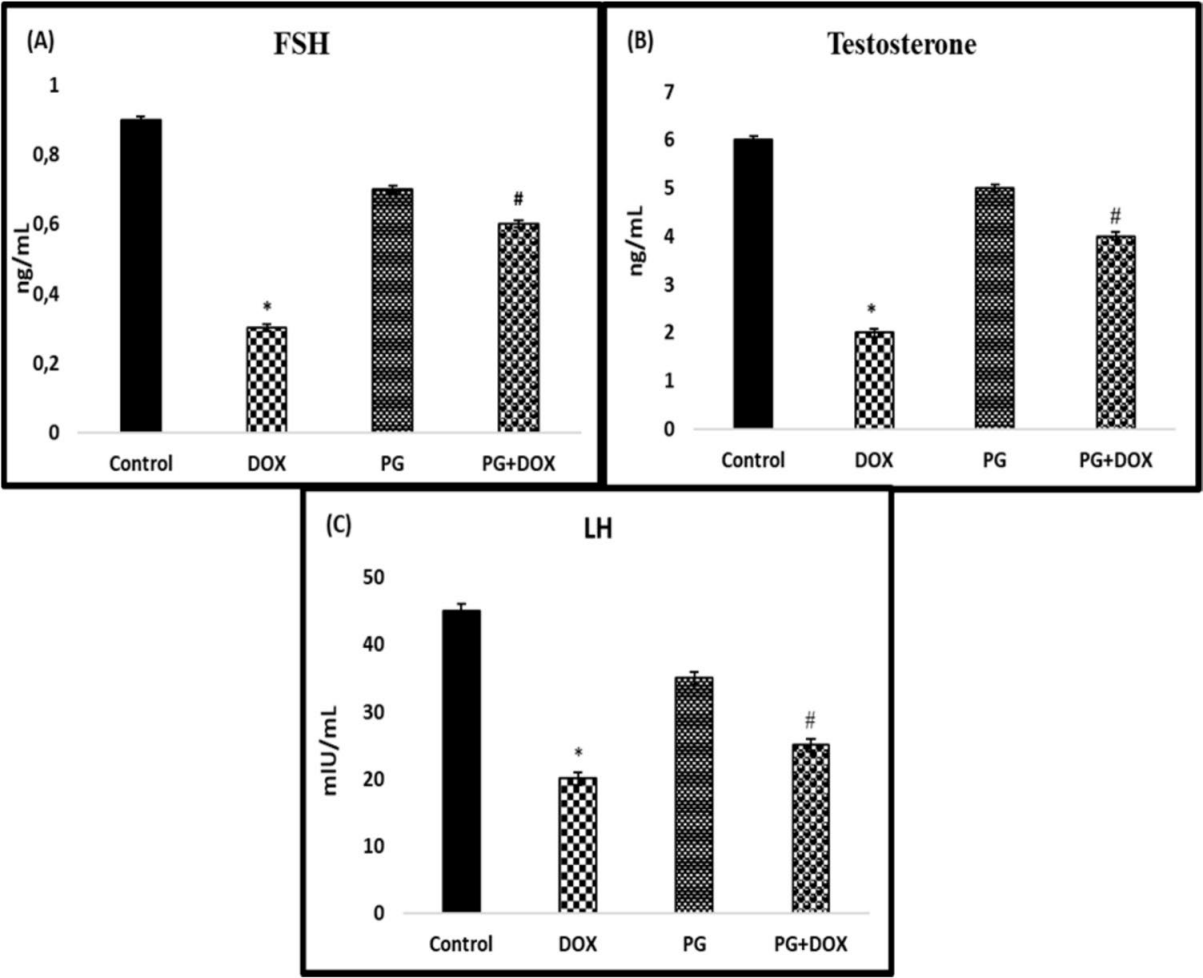
Effects of PG on serum (**A**) FSH, (**B**) testosterone, and (**C**) LH concentrations in experimental groups. *p < *0.05* versus. Control group, #*p* < *0.05* versus. DOX group (*n* = 8). Statistical analysis was done by one-way ANOVA followed by Tukey's LSD test. DOX: Doxorubicin, PG: 200 mg/kg *Panax ginseng*, DOX + PG: Doxorubicin + 200 mg/kg *Panax ginseng*

To determine the effect of DOX and PG on hormones, LH, FSH, and testosterone levels were evaluated in rat serum. Serum concentrations of sex hormones (LH, testosterone, and FSH) were significantly decreased in the DOX group compared to the control group (*p* < *0.05*). PG increased serum sex hormone concentrations compared to the DOX group. Serum sex hormone levels were significantly higher in the PG + DOX group than in the DOX group (*p* < *0.05*) (Fig. 4). PG treatment significantly improved the decreased sex hormone levels.

### COX-2, NF-κB, and AR Expression Levels in Testis Tissue

**Fig. 5:**
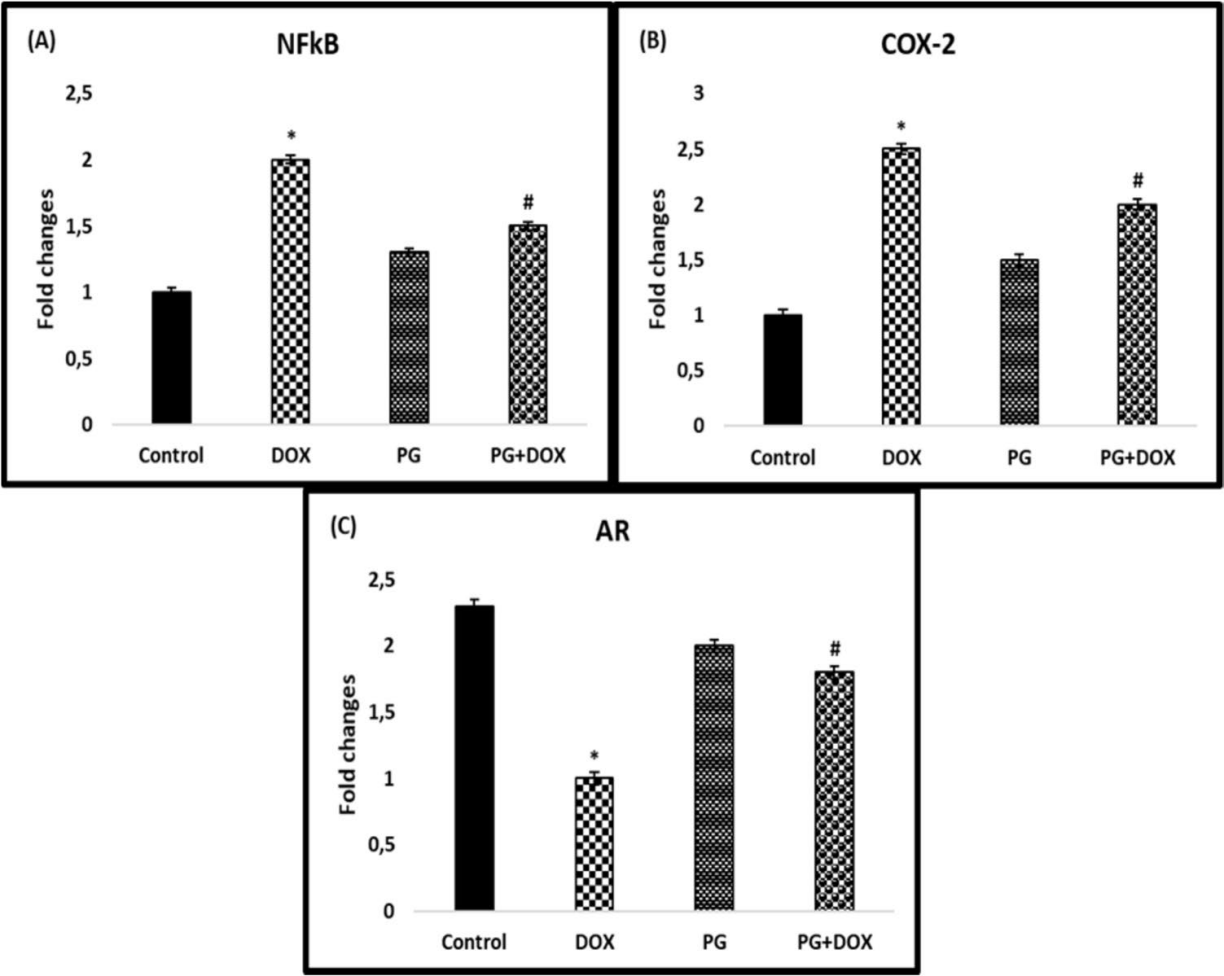
Relative gene expression levels of (A) Nf-κB, (B) COX-2, and (C) AR in the testis tissues of experimental groups. *p < *0.05* versus. Control group, #*p* < *0.05* versus. DOX group (*n* = 8). Statistical analysis was done by one-way ANOVA followed by Tukey's LSD test. DOX: Doxorubicin, PG: 200 mg/kg *Panax ginseng*, DOX + PG: Doxorubicin + 200 mg/kg *Panax ginseng*

COX-2, NF-κB, and AR were quantified by real-time PCR in testicular tissue to explain the possible mechanism underlying the above findings. While NF-κB expression was significantly upregulated in the DOX group compared to the control group (*p* < *0.05*), it was significantly downregulated in the PG + DOX group compared to the DOX group (*p* < *0.05*) (Fig. 5).

As shown in Fig. 5, COX-2 expression levels were significantly higher in the DOX group than in the control group (*p* < *0.05*), but significantly reduced in the PG + DOX group compared to the DOX group.

AR expression was significantly decreased in the DOX group compared to the control group (*p* < *0.05*). PG increased AR expression compared to the DOX group. AR expression was significantly higher in the PG + DOX group than in the DOX group (*p* < *0.05*) (Fig. 5).

### Histopathological Results

A statistically significant difference was found between the groups in the histopathological examinations (Table 2*, p* < *0.05*).

**Table 2:** Effect of PG treatment on MTBS levels in DOX-administrated rats

Groups	MTBS
Control	8,66 ± 0,75^a^
DOX	6,62 ± 0,40^b^
PG	8,45 ± 0,81^a^
PG + DOX	7,50 ± 0,30^c^

For statistical analysis, the Kruskal–Wallis test was used (*n* = 8). ^a,b,c^ show the difference between groups (*p* < *0.05*). While the groups indicated with the same letter do not show statistical significance with each other, there is statistical significance at the level of *p* < *0.05* between the groups with different letters. DOX, Doxorubicin; PG, 200 mg/kg *Panax ginseng*; DOX + PG, Doxorubicin + 200 mg/kg *Panax ginseng*

**Fig. 6:**
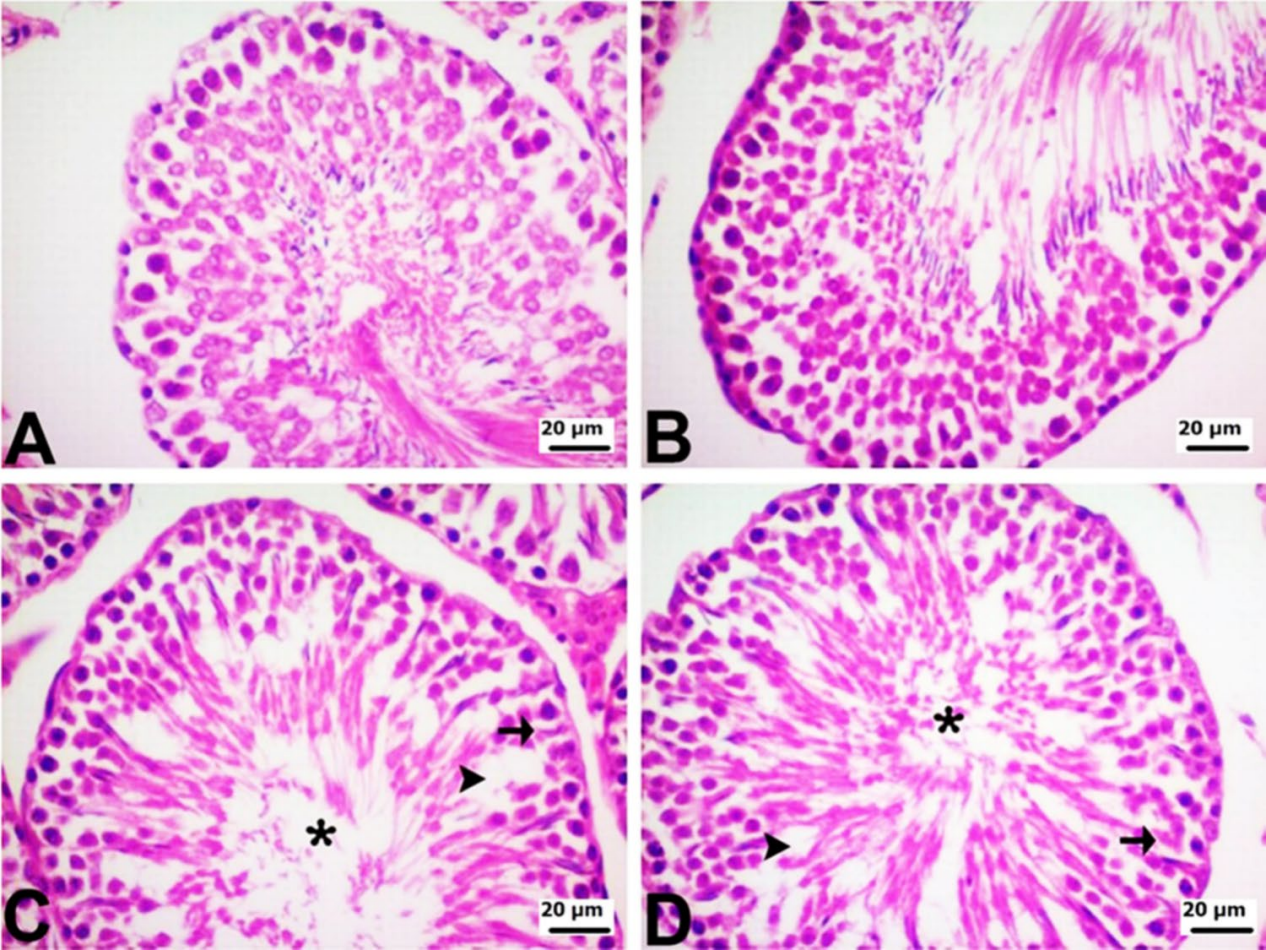
Histopathological appearance of testis sections (hematoxylin & eosin, × 40). **A** Control and **B** PG group normal histological appearance. **C** DOX group moderate degeneration (*), vacuolization (arrowhead) in seminiferous tubules, and degenerative changes (arrow) in spermatogenic cells. **D** DOX + PG group mild degeneration in seminiferous tubules (*), vacuolization (arrowhead), and degenerative changes in spermatogenic cells (arrow). DOX group. DOX: Doxorubicin, PG: 200 mg/kg *Panax ginseng*, DOX + PG: Doxorubicin + 200 mg/kg *Panax ginseng*

Hematoxylin and eosin staining was performed to evaluate histopathological changes in spermatogenic cells in seminiferous tubules. The testes of rats in the control and PG groups had a normal histological appearance. On the other hand, moderate degenerative changes and vacuolization were observed in spermatogenic cells in the seminiferous tubules in the DOX group. Degenerative changes and vacuolization were also milder in spermatogenic cells in seminiferous tubules in the PG + DOX group compared to the DOX group (Fig. 6). In the DOX group, only a small number of early spermatids were observed within the seminiferous tubules. However, following PG administration, spermatogenic cell development in seminiferous tubules improved in the DOX group. Although not fully differentiated, early spermatids constituted the majority of germ cells. Additionally, the seminiferous tubules exhibited a more uniform structural appearance.

### Immunohistochemical Results

There was a significant difference between the groups regarding AR, COX-2, and NF-κB immunopositivity (Table 3, *p* < *0.05*).

**Table 3:** Scoring of immunopositivity in immunohistochemical staining with AR, COX-2, and NF-κB in testicular tissue

Groups	AR	COX-2	NF-κB
Control	2,83 ± 0,40^a^	0,83 ± 0,40^a^	0,83 ± 0,40^a^
DOX	1,16 ± 0,40^b^	2,66 ± 0,51^b^	3,66 ± 0,51^b^
PG	2,83 ± 0,40^a^	1,00 ± 0,40^a^	0,83 ± 0,40^a^
PG + DOX	2,00 ± 0,00^c^	1,83 ± 0,40^c^	2,16 ± 0,40^c^

For statistical analysis, the Kruskal–Wallis test was used (*n* = 8). ^a,b,c^ show the difference between groups (*p* < *0.05*). While the groups indicated with the same letter do not show statistical significance with each other, there is statistical significance at the level of *p* < *0.05* between the groups with different letters. DOX, Doxorubicin; PG, 200 mg/kg *Panax ginseng*; DOX + PG, Doxorubicin + 200 mg/kg *Panax ginseng*

**Fig. 7:**
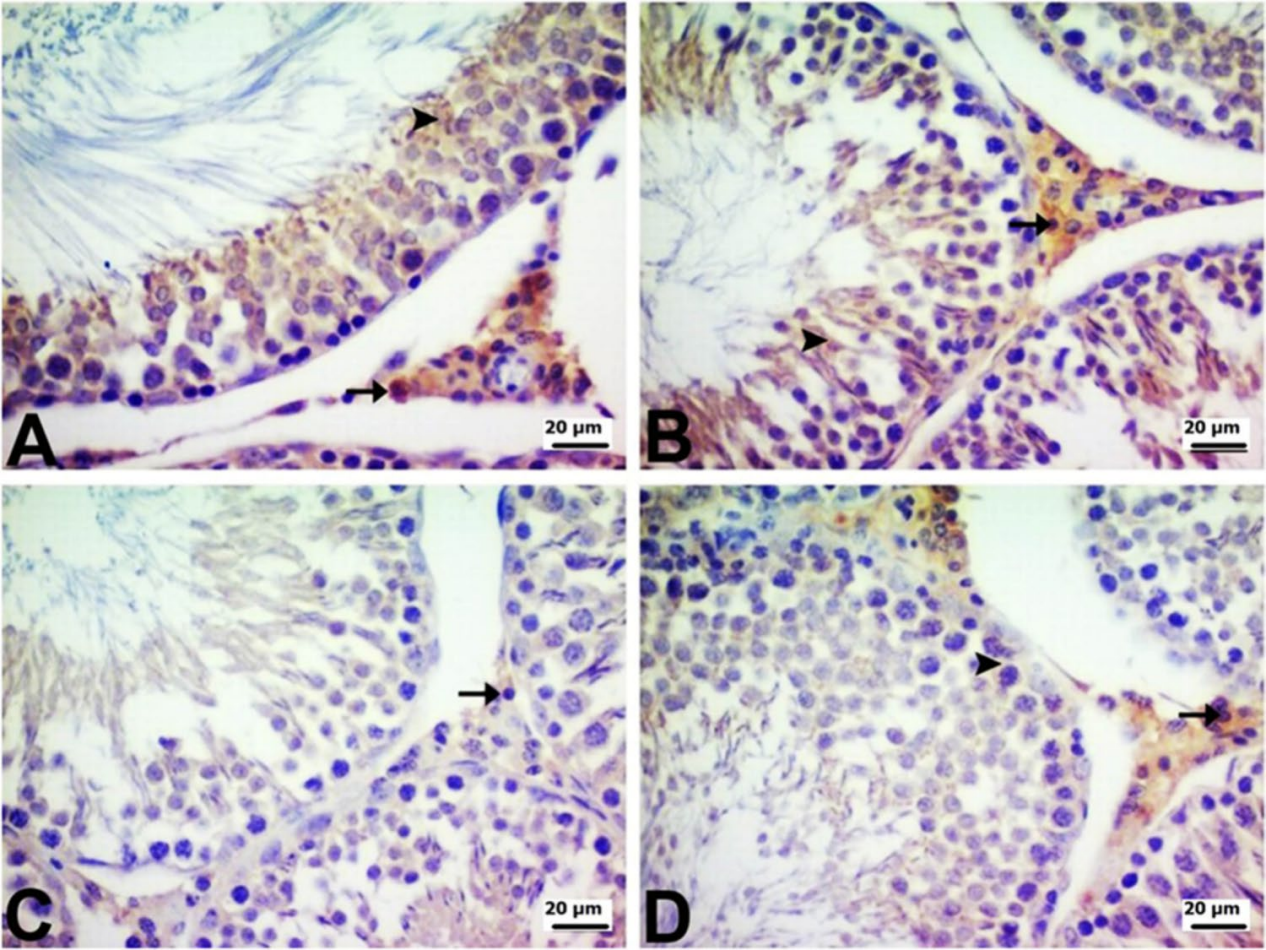
**A** Control and **B** PG groups show severe intractoplasmic levels in Leydig cells (arrow) and spermatogenic cells (arrowhead). **C** DOX group mild intractoplasmic in Leydig cells (arrow). **D** PG + DOX group moderate intractoplasmic AR immunopositivity in Leydig cells (arrow) and mild intractoplasmic in spermatogenic cells (arrowhead). DOX: Doxorubicin, PG: 200 mg/kg *Panax ginseng*, DOX + PG: Doxorubicin + 200 mg/kg *Panax ginseng*

**Fig. 8:**
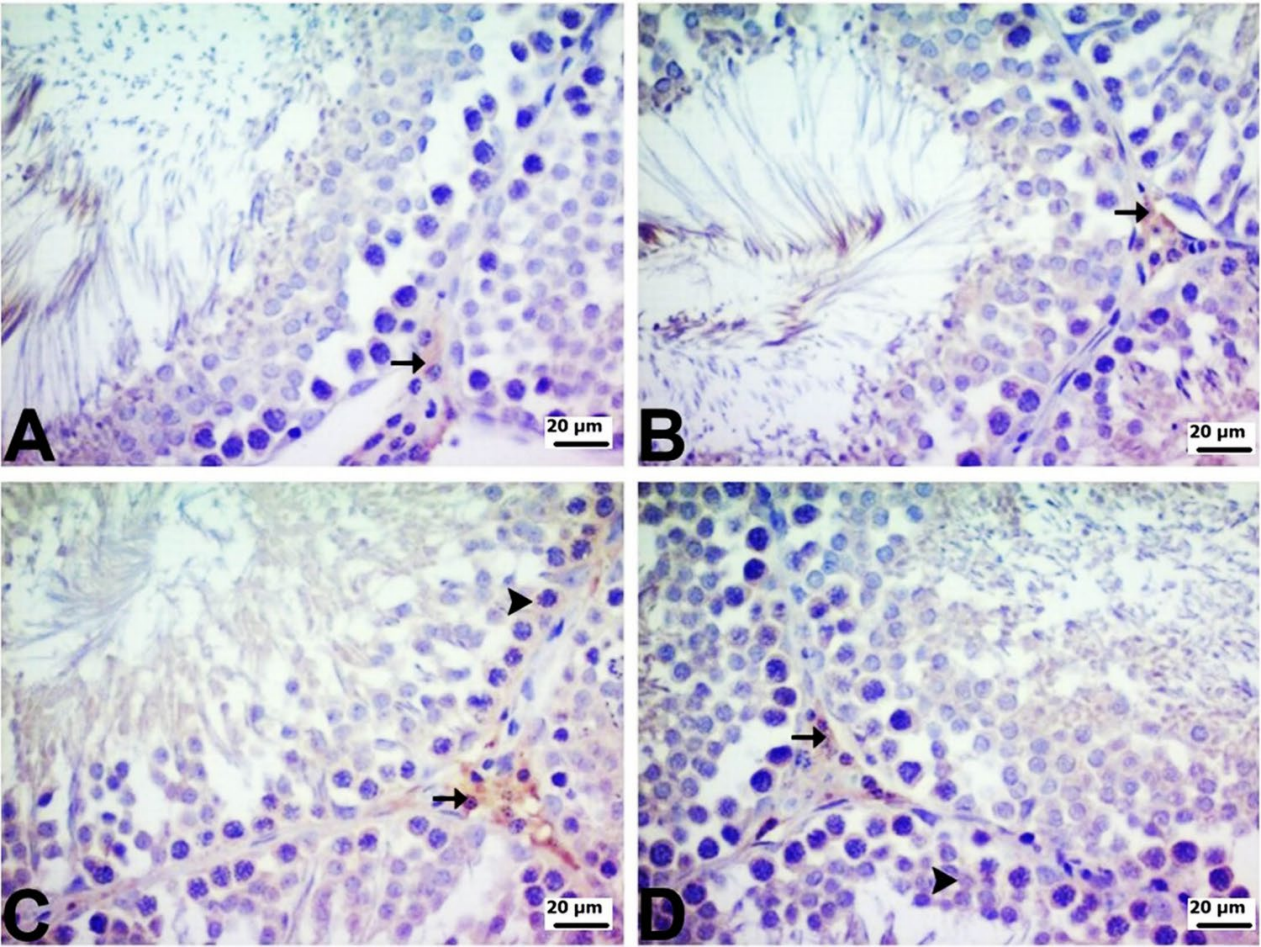
**A** Control and **B** PG group mild intractoplasmic in Leydig cells (arrow). **C** DOX group: severe intractoplasmic in Leydig cells (arrow), moderate intractoplasmic in spermatogenic cells (arrowhead). **D** PG + DOX group moderate intractoplasmic COX-2 immunopositivity in Leydig cells (arrow) and mild intractoplasmic in spermatogenic cells (arrowhead). DOX: Doxorubicin, PG: 200 mg/kg *Panax ginseng*, DOX + PG: Doxorubicin + 200 mg/kg *Panax ginseng*

**Fig. 9:**
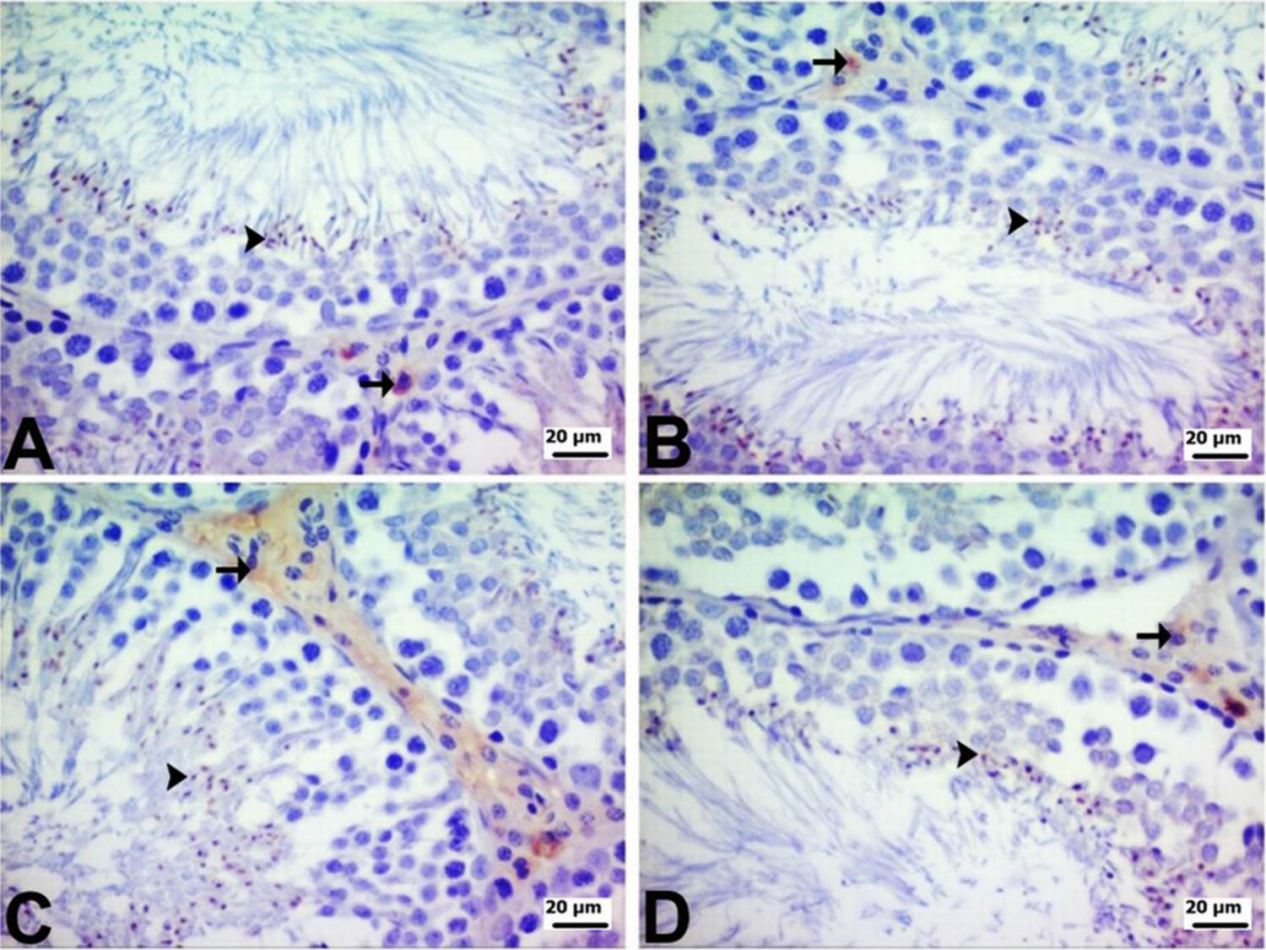
**A** Control and **B** PG groups were mild intractoplasmic in Leydig cells (arrow) and mild intranuclear spermatids (arrowhead), **C** DOX group is very severe intractoplasmic in Leydig cells (arrow) and mild intranuclear spermatid (arrowhead), **D** PG + DOX group moderate to mild intractoplasmic NF-κB immunopositivity in Leydig cells (arrow) and mild intractoplasmic spermatids (arrowhead). DOX: Doxorubicin, PG: 200 mg/kg *Panax ginseng*, DOX + PG: Doxorubicin + 200 mg/kg *Panax ginseng*

COX-2, NF-κB and AR immunopositivity in testicular tissue was determined by immunohistochemical staining. AR immunopositivity was strong, whereas COX-2 and NF-κB immunopositivity was mild in the testes of rats in the control and PG groups. On the other hand, there were differences in AR, COX-2, and NF-κB immunopositivity in the DOX and PG + DOX groups. AR immunopositivity was mild in the DOX group and moderate in the PG + DOX group. COX-2 immunopositivity was strong in the DOX group and moderate in the PG + DOX group. Similarly, NF-κB immunopositivity was very severe in the DOX group and moderate in the PG + DOX group. AR, COX-2, and NF-κB immunopositivity were seen mainly in Leydig cells in the interstitial region and to a lesser extent in spermatogenic cells and spermatids. When AR, COX-2, and NF-κB were compared, AR generally exhibited the strongest immunopositivity. In terms of localization, immunopositivity was observed to cover the entire structure of the spermatids, whereas in Leydig cells and spermatogenic cells, it was localized intracytoplasmically. The intranuclear immunopositivities observed in spermatids were mild, while the intracytoplasmic immunopositivities in Leydig and spermatogenic cells varied from mild to intense (Fig. 7, 8, and 9).

## Discussion

The rising incidence of malignancies in the world is responsible for the frequent prescription of anticancer medications. So, it is crucial to protect male fertility against the negative consequences of the usage of these drugs [1]. PG has many beneficial properties, including anti-inflammatory, anticancer, hypoglycaemic, and antioxidant properties [20, 28, 29]. The current study investigated the possible alleviating potential provided by PG in a DOX-induced testicular injury model. The study showed improved serum sex hormone levels, inflammation, and oxidative load. In addition, the results revealed that PG maintains testicular histological and gene (NF-κB, AR, and COX-2) changes against DOX-induced testicular damage. It paralleled the protective effect of PG, consistent with previous studies [30, 31]. To the authors'knowledge, no literature study has examined the potential beneficial effects of PG on DOX-induced testicular damage in rats.

In this study, LDH, CAT, GSH, MDA, and SOD activities were measured to assess oxidative stress, which plays a significant role in male infertility caused by the toxic effects of DOX. DOX-induced free radical generation triggers lipid and protein oxidation and depletes antioxidant defense systems (SOD, CAT, and GSH). Complementary oxidative stress and the onset of inflammation play a significant role in DOX-induced testicular damage [32, 33]. In toxicity studies with various chemotherapeutic agents, inflammatory parameters have increased in testicular tissue [34, 35]. However, pre-treatment with PG alone prevented and minimized the detrimental effects in DOX-treated rats. On the other hand, IL-1β and TNF-α levels were also measured to assess DOX-induced pro-inflammation in the testes. In line with the results obtained, DOX significantly increased IL-1β and TNF-α levels, while PG reduced inflammatory parameters. This study is consistent with previous studies reporting that PG has anti-inflammatory properties [36].

Another important phenomenon that threatens male fertility in DOX-induced testicular toxicity is the decrease in testosterone levels [37, 38]. Androgens play an important role in maintaining spermatogenesis, male reproductive differentiation, and enhancing sexual characteristics [39]. Most of the activities of androgens (LH and testosterone) are regulated by AR in target cells [40]. Consistent with the present study, previous reports have shown that chemotherapeutic drugs reduce serum testosterone levels and AR expression in the testis [41]. DOX administration reduced both serum LH and testosterone levels and AR expression. It is suggested that reduced AR expression and serum LH levels may contribute to reduced testosterone secretion in Leydig cells [40]. This work showed that PG treatment upregulated AR expression and reversed FSH, LH, and testosterone levels. Androgens, which require androgen receptors, play a key role in maintaining epididymal sperm maturation and regulating testicular spermatogenesis [42]. Therefore, the degradation of the androgen mechanism also negatively affects spermatogenesis. LH enables Leydig cells to secrete testosterone, resulting in negative feedback that suppresses LH secretion [43]. The present study has shown that the reduction in LH levels in DOX-treated rats can lead to hypopituitarism and decrease the sensitivity of the hypothalamic-pituitary axis to testosterone.

Blocking the COX-2 and NF-κB pathways is one of the major strategies to control inflammation. The transcription factor NF-κB is required for the synthesis of COX-2 protein. Signals that activate NF-κB induce COX-2 expression because they contain the promoter site of the COX-2 gene, which is a binding region for the p65 subunit of NFκB-3 [32, 44]. The synthesized COX-2 protein releases prostaglandins, which can cause severe inflammation [45]. Many studies have shown that PG reduces the production of inflammatory mediators [20, 28, 36]. The results of the current study support previous studies and show that PG can reduce inflammation through its effect on inflammatory mediators and antioxidant activity. Data obtained from immunohistochemistry and real-time PCR analyses showed that NF-κB and COX-2 expression increased and AR expression decreased in DOX-treated testicular cells compared to the control group. Therefore, it can be concluded that DOX-induced oxidative stress leads to an increase in NF-κB expression, which leads to increased expression of various genes, such as COX-2, causing inflammation in testicular cells. PG + DOX treatment resulted in the suppression of NF-κB and COX-2 and the induction of AR in rat testicular cells. PG suppressed DOX-induced oxidative stress and inflammation in rat testes. Histopathological analysis showed that the DOX group caused the degeneration and vacuolization of spermatogonia/spermatocytes. On the other hand, most of the pathologies observed in the DOX group were not observed in the PG-treated groups. Taken together, these data suggest that PG may exert or contribute to protective and therapeutic effects in DOX-induced testicular toxicity.

The current study had several limitations. Apoptotic markers, semen, epididymal tissue samples, and sperm kinematic values were not measured in the study. Furthermore, although our data suggest the therapeutic use of PG, future in-depth studies are required to evaluate whether PG affects the chemotherapeutic activity of DOX. In addition, it is recommended that the same interventions be performed on cancer rats and that different training protocols be evaluated.

## Conclusion

From the present study, it can be concluded that PG is one of the effects that protect against DOX-induced testicular toxicity in rats by reducing lipid peroxidation and activating the antioxidant system. PG may safeguard the reproductive health of male patients undergoing chemotherapy by offering a potential preventive treatment against DOX-induced testicular damage. Testicular damage may affect male reproductive health and lead to infertility. Such preventive therapies are of great clinical importance for patients concerned about infertility and individuals who want to preserve their testicular function during the treatment process. As a result, it may be beneficial for male patients undergoing cancer treatment. Further scientific studies are needed to investigate this relationship between PG and DOX and to elucidate its mechanisms.

## Data Availability

''The data supporting this work's findings are available from the corresponding author upon plausible demand.''.
